# Isolated Increased Nuchal Translucency With Normal Chromosomal Study: A Case Report

**DOI:** 10.7759/cureus.49209

**Published:** 2023-11-21

**Authors:** Nihar R Bhoi, Vipin Chandra, Kshitiz Murdia, Taruna Jhamb

**Affiliations:** 1 Reproductive Medicine, Indira IVF Hospital Private Limited, Udaipur, IND; 2 Clinical Research and Operations, Indira IVF Hospital Private Limited, Udaipur, IND

**Keywords:** fetal echocardiography, in vitro fertilization, hypoechoic swelling, single embryo transfer, intracytoplasmic sperm injection, cytogenetic analysis, genomic structural variation, nuchal translucency measurement

## Abstract

Increased nuchal translucency (NT) leads to a higher risk of fetal structural abnormalities. The measurement between 11 and 14 weeks gestation is a reliable marker for associated chromosomal abnormalities. Here, we present the case of a 33-year-old female with isolated high NT in the range of 5.6 mm at 12 weeks of gestational age. She was evaluated for chromosomal and structural abnormality and followed up meticulously. None of the tests showed any chromosomal or obvious structural abnormality. Fetal echocardiography revealed no structural cardiac defect. The pregnancy was uneventful and she delivered a healthy baby at term through lower (uterine)-segment cesarean section. The baby girl is living in good health without any developmental abnormalities. Although there is a high risk of chromosomal/structural defects with increased NT, it is not mandatory to terminate the pregnancy without a thorough evaluation.

## Introduction

Nuchal translucency (NT) is the sonographic form of a subcutaneous fluid collection behind the fetal neck in the first trimester of pregnancy [1]. NT is used regardless of the position, i.e., whether it is restricted to the neck or covers the entire fetus. The measurement of NT is done during 11 weeks to 12 weeks and six days and with a crown-rump length (CRL) of 45 mm to 84 mm [2]. Fetal abnormalities associated with increased NT may vary from 1.6% to 45%. Increased NT is strongly associated with adverse pregnancy outcomes, such as miscarriage, intrauterine death, congenital structural defects, and aneuploidy. When it is more than 99% of the expected, the prevalence of genetic disorders may vary from 0.5% to 6.6% [3]. A previous study observed that in fetuses with an increased NT, 17.8% had an adverse pregnancy outcome (miscarriage, intrauterine death, or termination for fetal abnormality) versus 1.5% for those with a normal measurement [4].

## Case presentation

A 33-year-old female from Rajasthan, India, conceived after seven years of primary infertility with her first attempt at in vitro fertilization (IVF) and intracytoplasmic sperm injection with single embryo transfer at Indira IVF Hospital, Udaipur. There was no medical history of any genetic disorder in either the maternal or paternal side of the family.

She was evaluated for chromosomal and structural abnormalities and followed up meticulously. None of the tests showed any chromosomal or obvious structural abnormality. Fetal echocardiography revealed no structural cardiac defect. Her pulse rate, blood pressure, hemoglobin, platelet counts, and hematocrit were within normal limits.

Her first trimester was uneventful. The first ultrasound examination in the sixth week showed a fetal pole with cardiac activity and a yolk sac. A follow-up ultrasound scan in the ninth week showed no gross fetal abnormality. At the 12th week (CRL = 56 mm), the ultrasound examination showed increased NT measuring 5.6 mm, but the nuchal edema was extending up to the lumber area. The nasal bone was visible. There was no evidence of tricuspid regurgitation, and ductus venosus flow was normal. There was a single umbilical artery.

She was advised for fetal karyotype (because of her medical history) and targeted ultrasound examination scan at 20 weeks and fetal echocardiography at 22 weeks of gestation.

**Figure 1:**
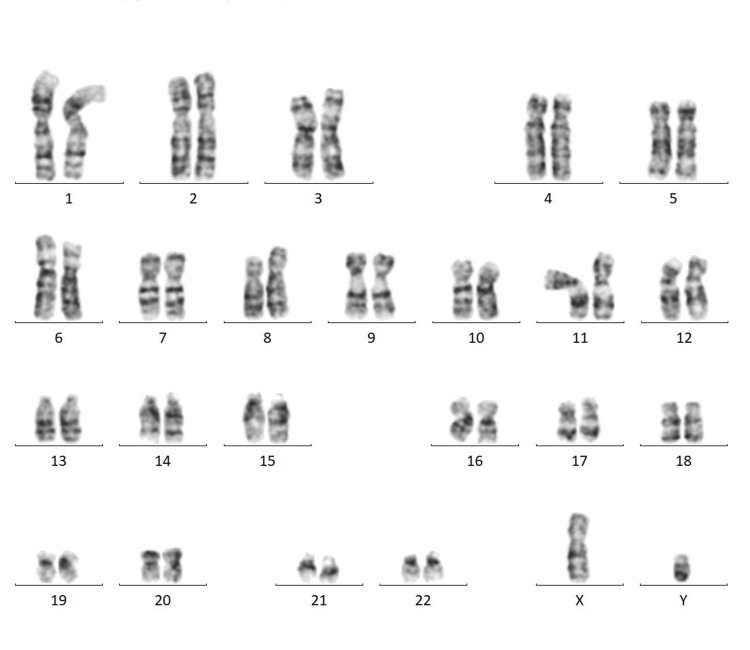
Karyotyping from amniocentesis specimen under GTG banding

**Figure 2:**
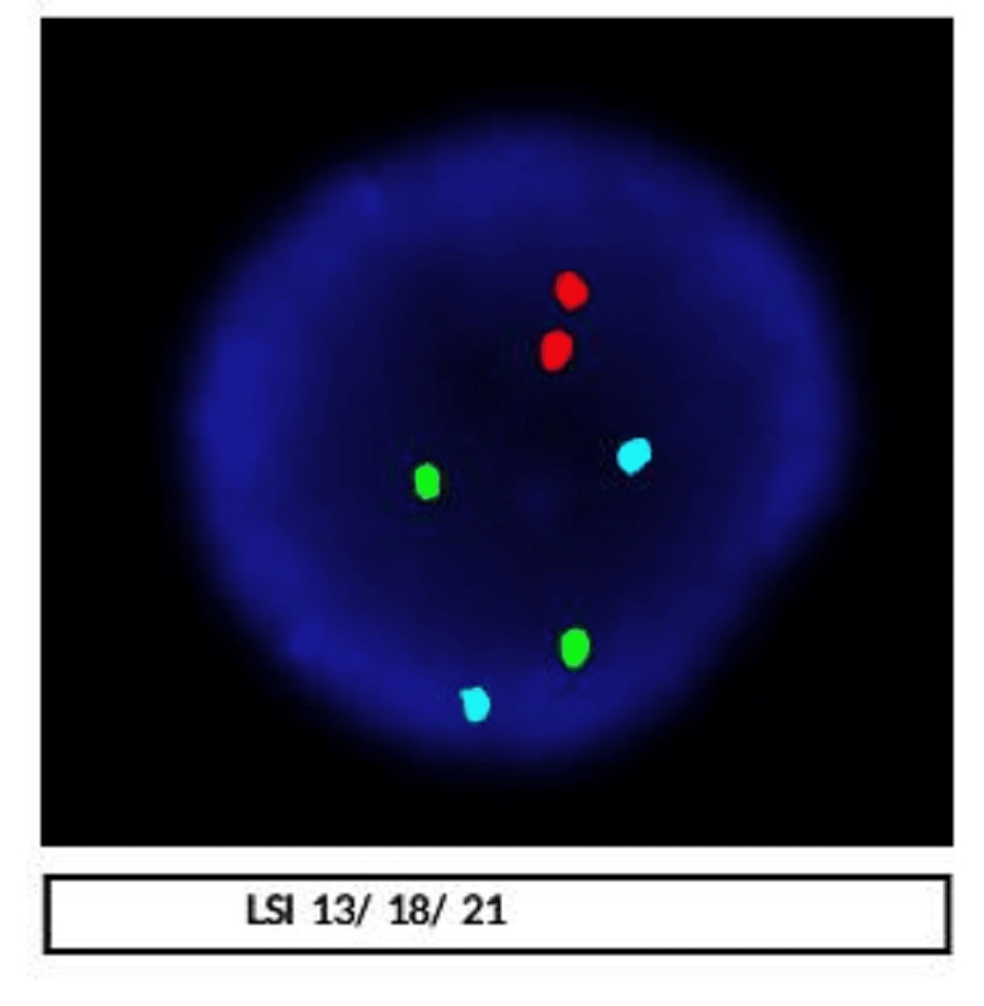
Chromosomes 13,18,21, X & Y were negative for deletion or duplication

Cytogenetic analysis by fluorescent in-situ hybridization for prenatal aneuploidy analysis for chromosomes 13, 18, 21, X and Y were negative for deletion or duplication. A total of 200 cells were scored for chromosomes 13, 18, 21 X and Y, of which 100% of cells were negative for the deletion or duplication of the given region (Figure 1). Overall, 46 chromosome complements were detected. No sex chromosomal abnormality was found at a resolution of 550 which did not detect any abnormality (Figure 2). At the 16th week of gestation, the scan showed a nuchal thickness of 4.6 mm, the nasal bone was visible, there was a single umbilical artery, and features were S/O cystic hygroma. The main features of cystic hygroma included a soft, spongy lump that commonly appeared on the neck. However, a cystic hygroma can also form in the armpits and groin area. The parents opted to continue the pregnancy after providing informed consent.

At the 20-week target scan, there was no apparent structural abnormality except a single umbilical artery. Nuchal fold thickness was 6.1 mm, and the nasal bone measured 6.3 mm. She was advised for fetal echocardiography at 22 weeks of gestational, which revealed no congenital heart defect and a single vertical pocket of liquor measuring 6.9 cm. The right lateral ventricle measured 6 mm, the left lateral ventricle measured 6.5 mm, and the cisterna magna measured 5.3 mm. The midline falx was seen. Both lateral ventricles appeared normal. The posterior fossa appeared normal with no identifiable intracranial lesion. The neck appeared normal. The entire spine was visualized in longitudinal and transverse axes. The vertebrae and spinal canal appeared normal. Both orbits, nose, and mouth appeared normal.

The rest of the pregnancy was uneventful. She delivered a term female baby by lower-segment cesarean section in March 2017. Post-delivery, microarray from the blood sample of the baby was found to have no chromosomal abnormality. Presently, she has completed four years of life without any developmental, physical, or psychological abnormality.

## Discussion

A heterogeneous group of conditions may be associated with increased NT. The pathology behind the accumulation of fluid in the soft tissue around the head and neck is due to venous congestion, the altered composition of the extracellular matrix, impaired lymphatic drainage, and, to some extent, fetal hypoproteinaemia and infection [5].

The major abnormalities detected in increased NT are skeletal defects, diaphragmatic hernia, cardiac defects, body stalk anomaly, Noonan syndrome, congenital adrenal hyperplasia, fetal akinesia, spinal muscular dystrophy, exomphalos, etc. There is a well-known close association between increased NT and fetal chromosomal defects [6]. This association allows the NT measurement to be converted into a likelihood ratio. Because the median NT increases with gestational age, the actual measurement must first be converted into a gestational age-specific multiple of median or a measurement of the deviation from the expected mean (delta NT) before conversion into a likelihood ratio [7].

Increased NT in fetuses is typically caused by genetic disorders and has a bad prognosis. However, with today’s standard prenatal diagnostic testing, more than 80% of such instances fail to yield a causal finding. As suggested by a study in fetuses with an NT ≥3.5 mm, prenatal chromosomal microarray should be offered. In a study by Lithner et al. [7], the overall risk for an adverse pregnancy outcome with an NT ≥3.5 and normal karyotype was 25.9%, indicating an overall chance of a favorable outcome of 74.1%. Petersen et al. [8], in their observation, suggested that the NT cut-off for invasive testing should be 3.0 mm (instead of 3.5 mm). However, a detailed scan without major structural defects can predict a favorable pregnancy outcome when chromosomal defects are ruled out [8].

In a previous study, in 55 cases (78%), there was no aneuploidy. A favorable pregnancy outcome at term was achieved in 40 cases (56% in total, 72% from euploid pregnancies). Additionally, pathogenic copy number variations (CNVs) accounted for just 0.8-5.3% of fetuses with isolated elevated NT (with/without other soft indicators), and some of these occurrences would have unfavorable outcomes. As a result, in this prenatal population, a test for the full identification of disease-associated genomic variations such as numerical abnormalities, structural rearrangements, CNVs, and point mutations is required [8].

## Conclusions

NT assessment should be advised in the first trimester. In case of enhanced NT with chromosomal abnormalities, the fetus should receive increased surveillance and specialized ultrasonography screening to establish the diagnosis. The report suggests that proper counseling should be done before the first scan, and all the test results should be discussed with transparency. Moreover, reassuring the parents and providing compassionate counseling until a proper evaluation is key to avoiding the termination of a normal fetus based on isolated increased NT.
